# High FAM189B Expression and Its Prognostic Value in Patients with Gastric Cancer

**DOI:** 10.1155/2021/8875971

**Published:** 2021-05-25

**Authors:** Chang-Liang Wu, Quan-Xiao Tan, Dan Liu, Jun-E Jiang, Ting Lu, Zu-Mei Huang, Ying-Jie Su

**Affiliations:** Department of Gastroenterology, Guangxi International Zhuang Medicine Hospital, Nanning, 530200 Guangxi, China

## Abstract

The clinical significance of the family with sequence similarity 189 member B (FAM189B) gene remains largely unknown in gastric cancer (GC). A comprehensive investigation combining multiple detection methods was carried out in the current study to unveil the clinical implications and prospective molecular characterization of FAM189B protein and mRNA in GC. The protein level of FAM189B was clearly upregulated in the tumor tissues of GC as compared to noncancerous gastric tissues with 179 GC cases and 147 noncancerous gastric controls assessed by immunohistochemistry. The upregulation of the FAM189B protein was also found in the more deteriorating period of the tumor, as there were increasing trends in the groups of larger tumors, with lymph node metastasis, a further advanced clinical stage, and a higher histological grade. Next, we focused on the mRNA level of FAM189B in GC tissues using various high-throughput data. After the screening of GEO, ArrayExpress, and SRA, we finally achieved 18 datasets, including an RNA sequencing dataset of TCGA. Altogether, 1095 cases of GC tissue samples were collected, with 305 unique examples of noncancerous controls. Concerning the mRNA level of FAM189B in GC, the final standard mean difference (SMD) was 0.46 and the area under the curve (AUC) was 0.79 for the upregulation of FAM189B mRNA, which confirmed that the FAM189B mRNA level was also markedly upregulated in GC tissues and comparable to its protein level. The survival analysis showed that the higher expression of FAM189B was a risk factor for the overall survival, first progression, and postprogression survival of GC. For the Affymetrix ID 1555515_a_at of FAM189B, the higher expression level of FAM189B predicted a lower overall survival, first progression survival, and postprogression survival with the hazard ratio (HR) being 1.56 (1.24, 1.95), 1.69 (1.32, 2.17), and 1.97 (1.5, 2.6), respectively. For the Affymetrix ID 203550_s_at of FAM189B, a similar result could be found with corresponding HR being 1.49 (1.24, 1.8), 1.49 (1.21, 1.83), and 1.66 (1.32, 2.08), respectively. The interaction of MEM, COXPRESdb coexpressed genes, and DEGs of GC finally generated 368 genes, and the pathway of the cell cycle was the top pathway enriched by KEGG. In conclusion, the overexpression of the FAM189B protein and mRNA might enhance the incidence of GC.

## 1. Introduction

Gastric cancer (GC) is one of the most common types of cancer in the world and one of the most common causes of cancer-related death [1–4]. Previous studies have shown that GC is a multifactorial cancer. It is also a disease that displays multiple stages. Among various pathogenic factors, environmental issues (particularly dietary influences and *Helicobacter pylori* infection) are considered to play a crucial role in the pathogenesis of GC. Unhealthy dietary habits can affect some molecular functions and promote the occurrence of GC [5–11]. Different markers also play corresponding roles in the development and deterioration of GC. At present, some molecular mechanisms of GC progression have been identified [12–24], but the molecular pathological mechanism of GC is complex; hence, there is still room for inquiry into the molecular machinery of GC. These potential molecular targets for tumorigenesis and progression will play an important role in the detection and diagnosis of GC and may even influence the development of targeted drugs.

Among the potential genes that remain blank in many studies, the family with sequence similarity 189 member B gene (FAM189B, also known as COTE1 or C1orf2) has only been reported by two research groups in hepatocellular carcinoma (HCC) [25, 26]. FAM189B is located adjacent to the gene for the lysosomal enzyme glucosylceramidase; an absence of this enzyme is associated with Gaucher disease. Few studies have been carried out on the clinical role and mechanism of FAM189B in malignant tumors, except in HCC. Both the FAM189B mRNA and protein levels are noticeably upregulated in HCC clinical samples and HCC cell lines [25, 26]. The overexpression of FAM189B was related to the presence of larger tumors and poorly differentiated tumor status [26]. Additionally, the ability of HCC cells to invade other tissues was enhanced after overexpressing FAM189B *in vitro* [25]. Experimental ectopic overexpression of FAM189B boosted *in vitro* HCC cell viability and colony formation and also the *in vivo* tumorigenicity of HCC-derived cells [26]. However, the expression level of FAM189B, regardless of the protein or mRNA levels, has not yet been investigated in GC. The clinical significance of FAM189B in GC therefore remains largely unknown.

A comprehensive investigation combining multiple detection methods was carried out in the current study to unveil the clinical implications and prospective molecular characterization of FAM189B in GC. The current findings may help clinicians better interpret the role of FAM189B in the carcinogenesis of GC and provide a new direction for the clinical application of GC.

## 2. Materials and Methods

### 2.1. Protein Expression Levels of FAM189B in GC Tissues Detected by Immunohistochemistry

First, we detected the protein expression level of FAM189B in GC tissues via immunohistochemistry (IHC). Altogether, 179 GC cases and 147 noncancerous gastric controls were provided by the tissue microarrays from Pantomics, Inc. (Richmond, CA), together with the relevant clinicopathological parameters, including age, gender, tumor differentiation, tumor size (T), lymph node status (N), distant metastasis (M), and clinical TNM stages. The anti-FAM189B antibody (at 1/500 dilution, Biorbyt Explore Bioreagents catalog number: orb2035, rabbit) was the primary antibody used in the IHC experiment, and its keyhole limpet hemocyanin-conjugated synthetic peptide was derived from the region aa161-240 of human FAM189B. The FAM189B expression level of each section was assessed according to the score combined the percentage of stained cells and the staining degrees. The percentage of stained cells among all cancerous or normal gastric epithelium cells was recorded as follows: 0: <5%; 1: 5–25%; 2: 25–50%; 3: 50–75%; and 4: >75%; and the staining degree of the positive cells was recorded as follows: 0: no staining; 1: light yellow or yellow; 2: brown; and 3: dark brown [27, 28]. The lowest possible score was 0, while the highest score was 12.

### 2.2. mRNA Expression Level of FAM189B in GC Tissues Detected Using a High-Throughput Technique

In this study, in addition to the detection of the FAM189B protein level in clinical samples, we also examined the level of FAM189B mRNA expression. We collected the FAM189B mRNA data detected by multiple high-throughput technologies, including gene microarrays and RNA sequencing data assessed by various platforms. High-throughput data of GC was screened from Sequence Read Archive (SRA), ArrayExpress, Gene Expression Omnibus (GEO), Oncomine, and published studies. The retrieval keywords were as follows: (gastric OR stomach OR gastrointestinal) AND (cancer OR carcinoma OR tumor OR adenocarcinoma). The inclusion criteria were as follows: first, the dataset had both experimental and control groups. Second, the FAM189B mRNA levels could be successfully extracted. Third, this study was designed to include only clinical human GC and control tissue samples. Cell lines and animal model samples were not considered. The mRNA expression matrix data of each dataset was achieved, and the FAM189B mRNA expression data were extracted and subsequently converted into log2 for standby. The Cancer Genome Atlas (TCGA) and Genotype-Tissue Expression (GTEx) RNA sequencing data were routinely included as an independent dataset. TCGA and GTEx included the RNA sequencing data of 373 GC cases and 32 noncancerous gastric tissues. We also downloaded the clinicopathological parameters of each dataset, including sex, age, tumor grade, clinical TNM stage, and survival-related data, and further analyzed the relationships between FAM189B mRNA and the clinical progress of GC patients.

### 2.3. Integrated Investigation of FAM189B mRNA Expression Combining All Available Datasets

The standard mean difference (SMD) was calculated with Stata 12.0 software to show the comprehensive expression level of FAM189B based on all available data globally. Furthermore, the summarized receiver operating curve (sROC) was integrated using Stata 12.0, as well as the sensitivity and specificity of FAM189B expression.

### 2.4. The Relevant Signaling Pathways of FAM189B in GC

The FAM189B coexpressed genes in GC were downloaded from the Multi Experiment Matrix (MEM, *P* < 0.05) and COXPRESdb (the top 2000 genes). These FAM189B coexpressed genes were then interacted with the differentially expressed genes (DEGs) from the largest cohort with high-throughput data (e.g., TCGA and GTEx as assessed using the edgeR package). A log (fold change) of 1 and *P* < 0.05 were regarded as the cut-off values to select DEGs. The overlapped genes from MEM, COXPRESdb, and TCGA/GTEx were sent for the signaling pathway and protein-protein interaction (PPI) analyses. The mRNA and protein levels of several coexpressed genes were validated using TCGA/GTEx and the Human Protein Atlas (THPA) data.

### 2.5. Statistical Analyses

To analyze the variations of FAM189B expression levels in GC and noncancerous gastric tissues, Students' independent or paired sample *t*-tests were carried out when necessary using the Statistical Package for the Social Sciences (SPSS), version 19.0, including in-house IHC data, GC microarray data, and RNA sequencing data. A receiver operating curve (ROC) was generated to reveal the distinguishing ability of FAM189B expression to separate cancerous and noncancerous tissues. One-way analysis of variance was selected to analyze the differences of FAM189B expression among three or more groups of data. Lastly, the correlations between FAM189B and any other coexpressed genes were assessed using the Pearson correlation. The prognostic value of FAM189B was assessed by the Kaplan-Meier analysis, and the hazard ratio (HR) was also calculated. *P* < 0.05 was regarded as statistically significant.

## 3. Results

### 3.1. The Clinical Implication of the FAM189B Protein Level in GC Tissues Based on In-House Tissue Microarrays

**Figure 1:**
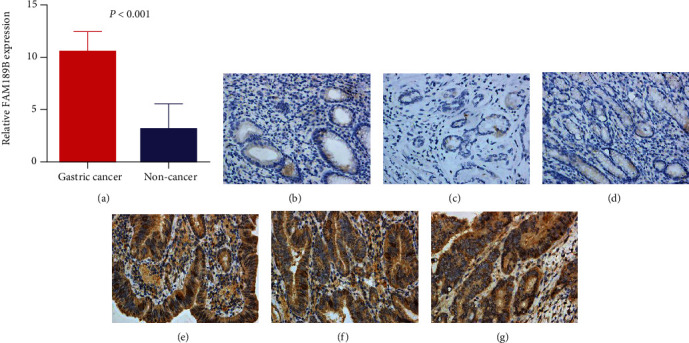
The protein expression level of FAM189B in gastric cancer tissues. In-house immunohistochemistry (IHC) was performed to compare the protein level of FAM189B between gastric cancer and noncancerous gastric tissues. IHC images from three cases are shown as examples. (a) IHC scores; (b–d) noncancerous gastric tissues; (e–g) gastric cancers (3,3′-diaminobenzidine (DAB) staining, ×400). Case 1 (b, e); case 2 (c, f); case 3 (d, g).

The protein level of FAM189B was clearly better upregulated in the tumor tissues of GC than in noncancerous gastric tissues (Figure 1, Table 1). The upregulation of the FAM189B protein was also found in the more deteriorating period of the tumor, as there were increasing trends in the groups of larger tumors, with lymph node metastasis, a further advanced clinical stage, and a higher histological grade (Table 1).

### 3.2. The FAM189B mRNA Level in GC Tissues Based on Various High-Throughput Data

**Figure 2:**
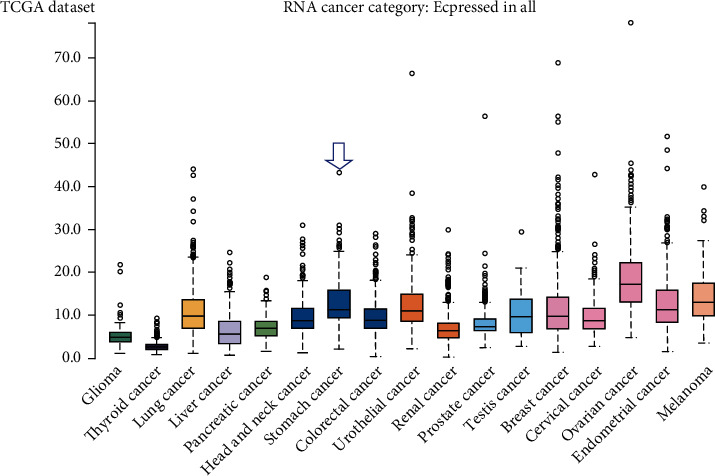
The mRNA expression level of FAM189B of pancancers based on RNA sequencing data. The mRNA expression level of FAM189B is presented as fragments per kilobase of exon per million reads mapped (FPKM) based on the Cancer Genome Atlas (TCGA). RNA sequencing data were calculated by the Human Protein Atlas (THPA) (https://www.proteinatlas.org/ENSG00000160767-FAM189B/pathology). Blue arrow: expression level of FAM189B mRNA in gastric cancer.

**Figure 3:**
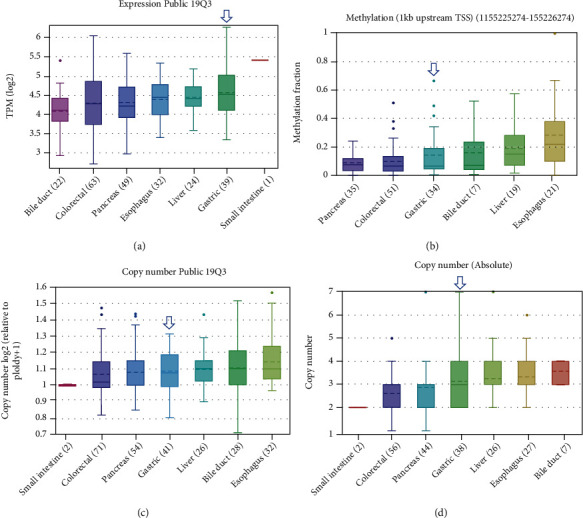
The molecular characteristics of FAM189B in pangastrointestinal cancer cell lines based on RNA sequencing data. (a) The mRNA expression level of FAM189B from Public 19Q3. (b) Methylation; copy number of FAM189B from Public 19Q3 (c) and Absolute (d). Blue arrow: FAM189B mRNA in gastric cancer. The different molecular characteristics of FAM189B in pangastrointestinal cancer cell lines were achieved from Cosmic (https://cancer.sanger.ac.uk/cosmic/gene/analysis?ln=FAM189B).

**Figure 4:**
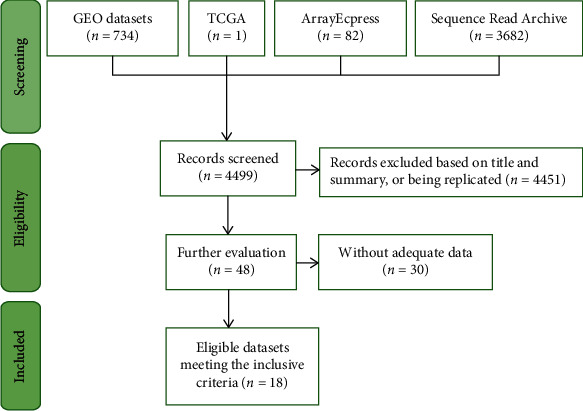
The screening flowchart to collect relatively high-throughput datasets of FAM189B mRNA expression in gastric cancer. GEO: Gene Expression Omnibus; TCGA: the Cancer Genome Atlas.

We further investigated whether the mRNA level of FAM189B was consistent with the protein level. First, the FAM189B mRNA was expressed to different degrees in the cancer tissues (Figure 2) and cells (Figure 3(a)) of multiple malignancies. Methylation and variations of the gene copy number were also observed in pangastrointestinal cancer cell lines (Figures 3(b)–3(d)). Next, we focused on the mRNA level of FAM189B in GC tissues using various high-throughput data. After the screening of GEO, ArrayExpress, and SRA, we finally achieved 18 datasets, including an RNA sequencing dataset of TCGA. Altogether, 1095 cases of GC tissue samples were collected, with 305 unique examples of noncancerous controls (Figure 4).

**Figure 5:**
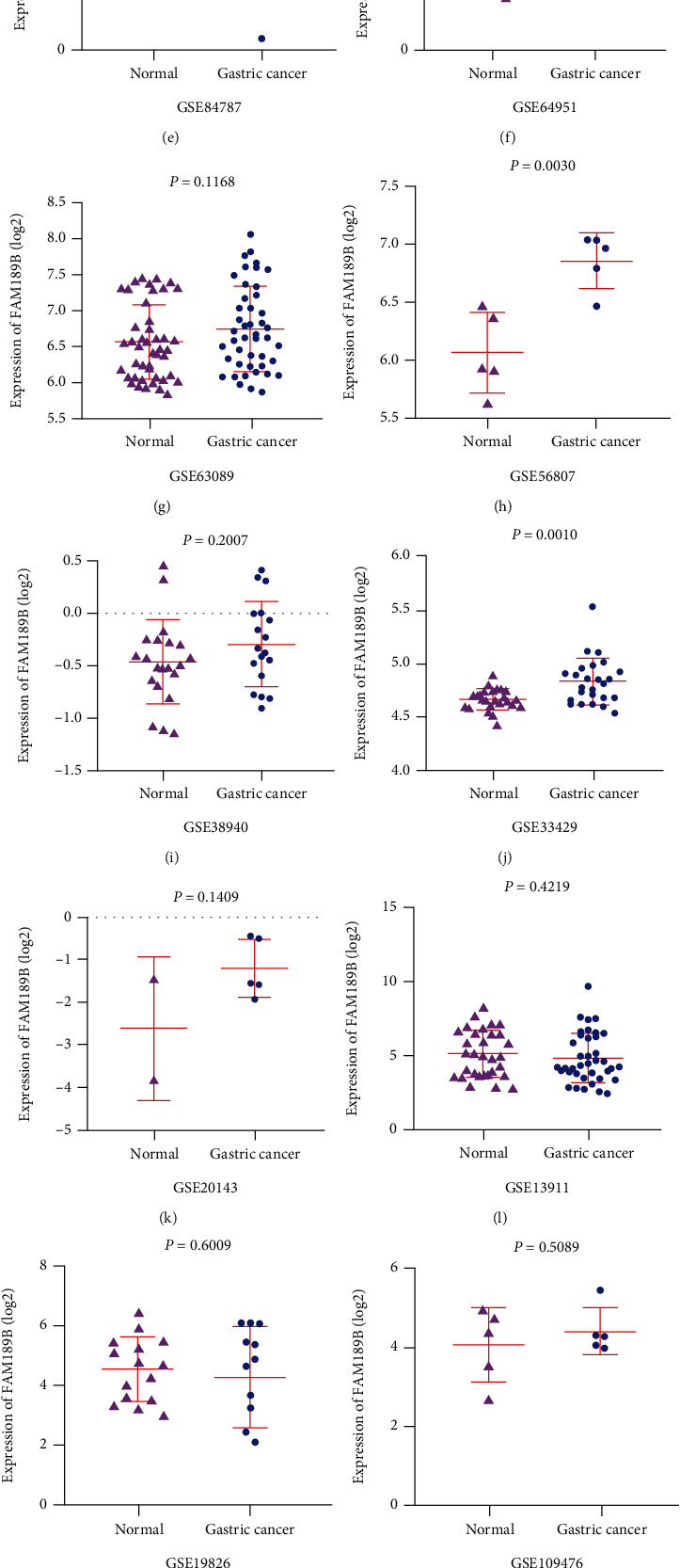
The FAM189B mRNA expression levels based on various high-throughput datasets in gastric cancer. The relative expression levels of FAM189B mRNA were extracted from different microarrays and RNA sequencing data. The scatter plots were drawn to compare the difference of FAM189B mRNA between noncancerous gastric tissues and gastric cancer.

**Figure 6:**
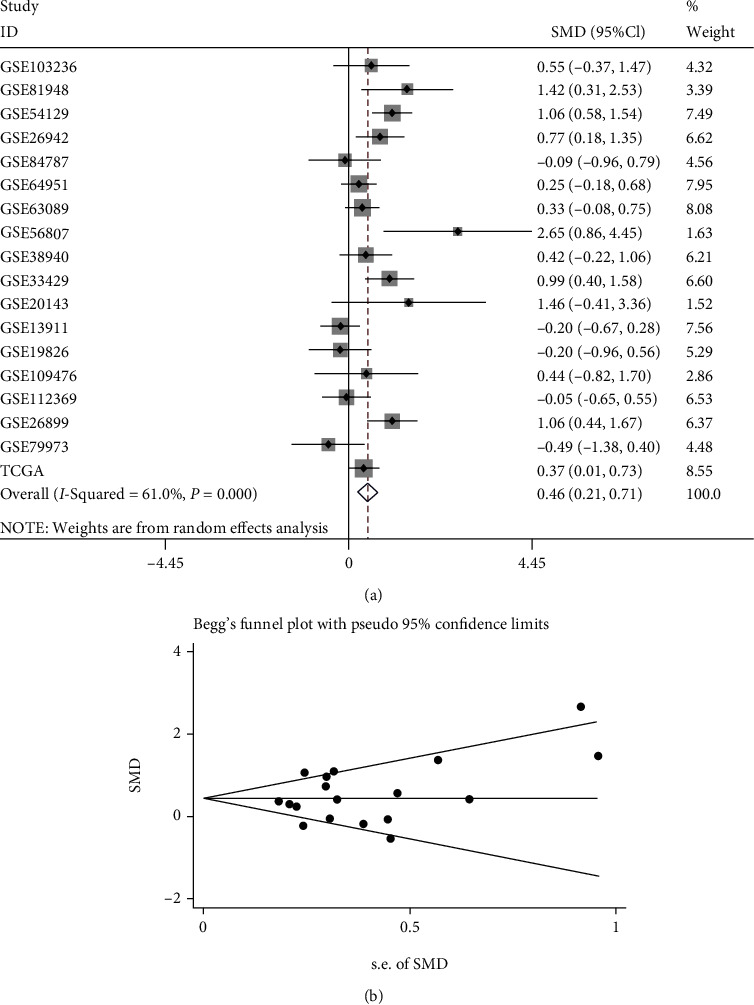
The integrated FAM189B mRNA expression level based on 18 high-throughput datasets in gastric cancer. The standard mean difference (SMD) was calculated for the integrated FAM189B mRNA expression level using a random effects model: (a) forest plot of the random effects model; (b) corresponding funnel plot.

**Figure 7:**
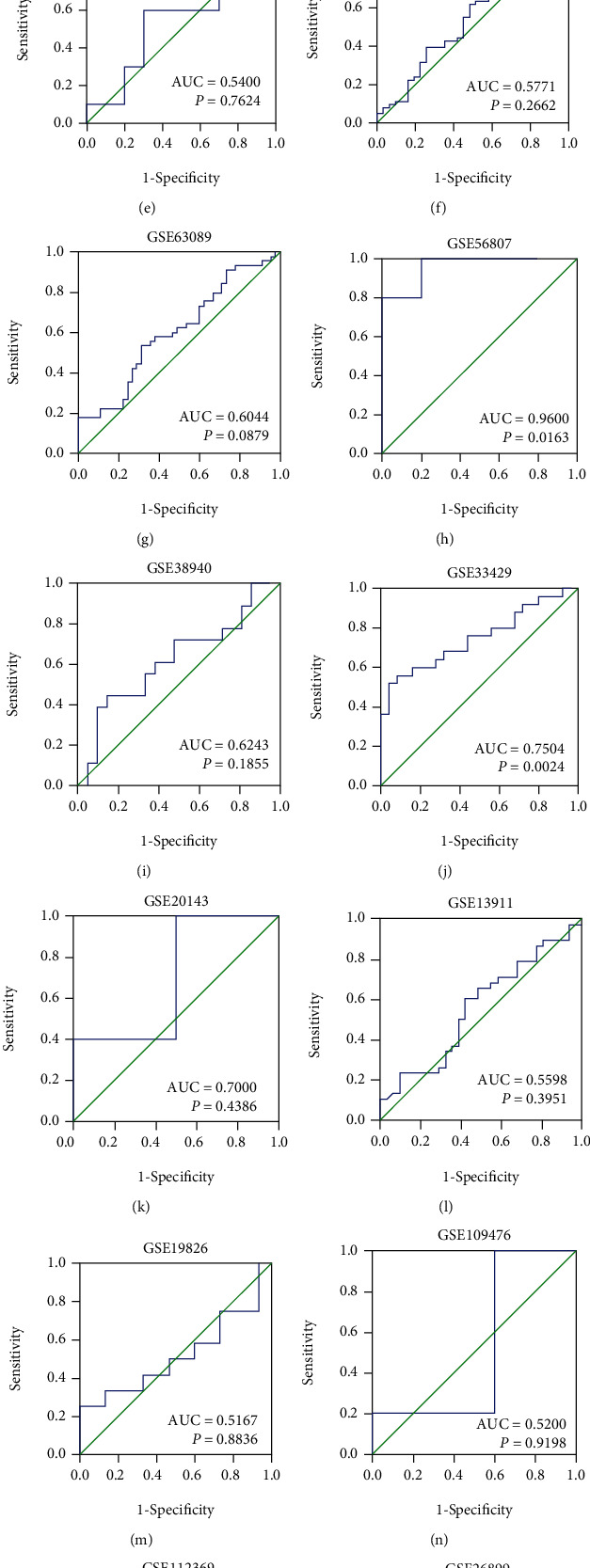
The area under ROC curves of FAM189B mRNA expression levels based on various high-throughput datasets in gastric cancer. The relative expression levels of FAM189B mRNA were extracted from different microarrays and RNA sequencing data in gastric cancer samples. The receiver operating characteristic (ROC) curves were assessed, and the area under the ROC curves (AUCs) was computed to compare the capacity of FAM189B mRNA to distinguish gastric cancer from noncancerous gastric tissues.

**Figure 8:**
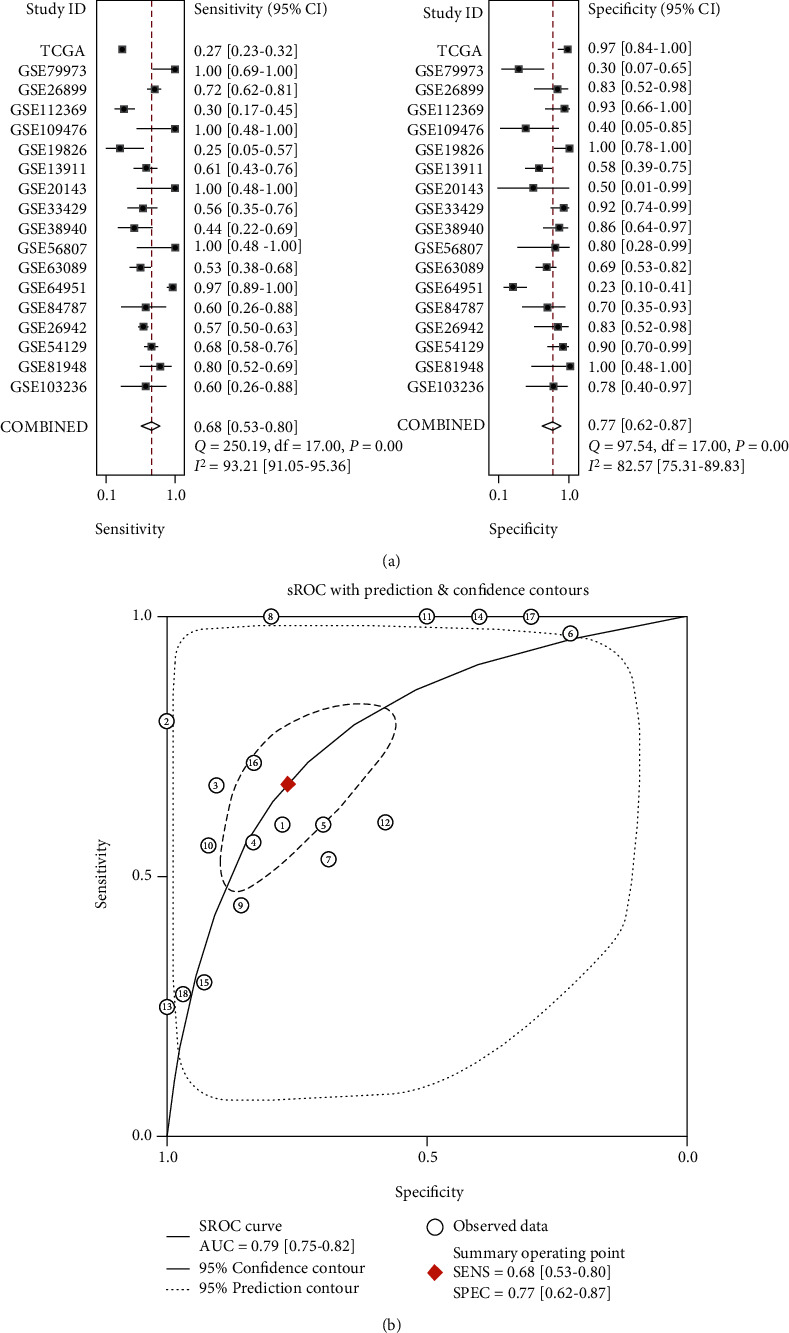
Integrated analysis to evaluate the ability of FAM189B to distinguish gastric cancer tissues from normal tissues: (a) the summarized ROC to access the potential of FAM189B to identify gastric cancer tissues and healthy tissues; (b) the pooled sensitivity and specificity.

Among 18 datasets, 7 studies showed that the mRNA level of FAM189B was significantly increased compared to the noncancerous controls (Figure 5). Since the difference of FAM189B mRNA between cancer and noncancer was quite inconsistent (Figure 5), a summarized SMD was calculated to provide an integrated image of the global mRNA expression of FAM189B based on all available microarrays and RNA sequencing data (Figure 6). The final SMD was 0.46 as analyzed by a random effects model (Figure 6), which confirmed that the FAM189B mRNA level was also markedly upregulated in GC tissues and comparable to its protein level, as previously mentioned. To further verify the upregulation of the FAM189B mRNA level in GC tissues, ROC curves were drawn for each dataset (Figure 7). Similarly, the areas under the curve (AUCs) varied in each study. To provide an overview of the distinguishing capacity of FAM189B mRNA in GC, we calculated the sROC (Figure 8(a)), which showed a moderate effect at 0.79. The sensitivity was 0.68, and the specificity was 0.77 for the FAM189B mRNA in GC (Figure 8(b)). These findings indicated that the overexpression of the FAM189B protein and mRNA might enhance the incidence of GC.

We were also interested in the role of FAM189B in the prognosis of GC. Two Affymetrix IDs (1555515_a_at and 203550_s_at) of FAM189B both showed that the higher expression level of FAM189B was a risk factor for the overall survival, first progression, and postprogression survival of GC [29]. The patient with high FAM189B expression showed a lower overall survival, first progression, and postprogression survival. For the Affymetrix ID 1555515_a_at of FAM189B, the higher expression level of FAM189B predicted a lower overall survival, first progression survival, and postprogression survival with the hazard ratio (HR) being 1.56 (1.24, 1.95), 1.69 (1.32, 2.17), and 1.97 (1.5, 2.6), respectively. For the Affymetrix ID 203550_s_at of FAM189B, a similar result could be found with the corresponding HR being 1.49 (1.24, 1.8), 1.49 (1.21, 1.83), and 1.66 (1.32, 2.08), respectively.

**Figure 9:**
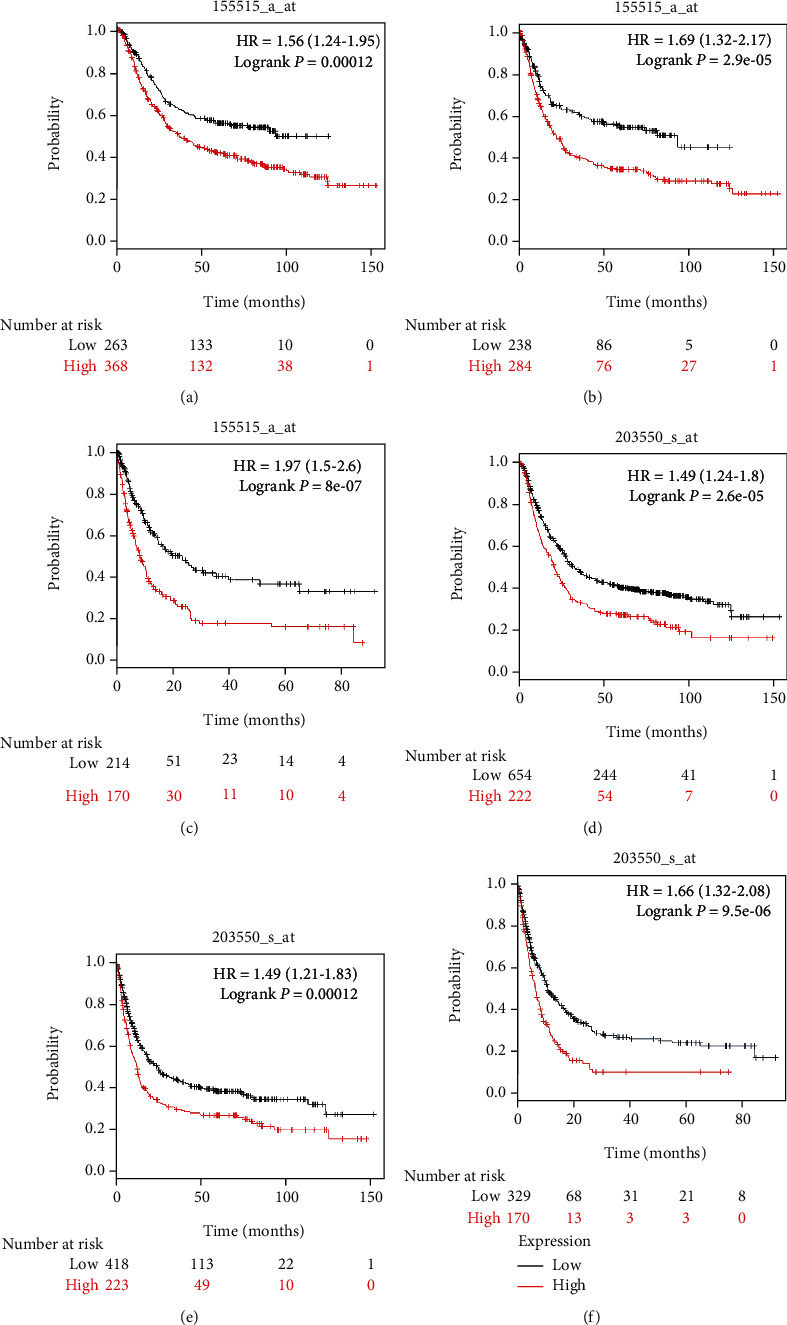
The prognostic value of FAM189B in gastric cancer: (a–c) dataset of 1555515_a_at; (d–f) dataset of 203550_s_at. Overall survival (OS) (a, d). First progression (FP). Postprogression survival (PPS) (c, f).

Since no sufficient datasets with survival information could be collected for the calculation of a pooled HR, the prognostic value of FAM189B will need to be validated in the future (Figure 9).

### 3.3. Related Signaling Pathways of FAM189B Coexpressed Genes in GC

**Figure 10:**
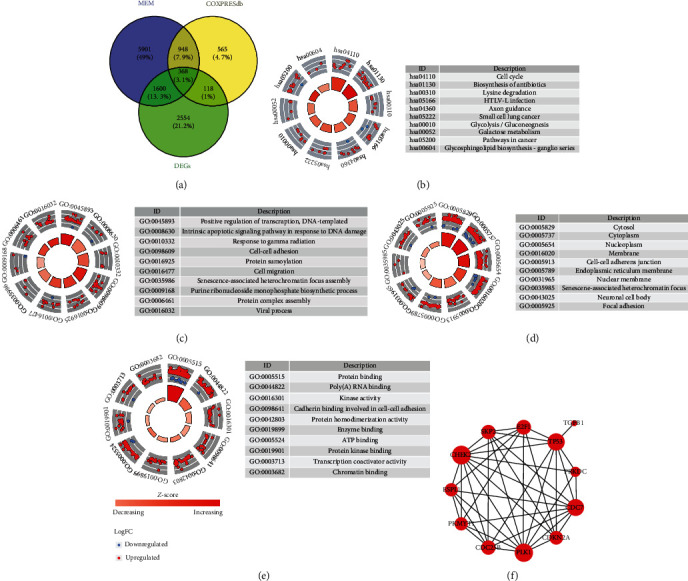
The relevant signaling pathways of FAM189B coexpressed genes in gastric cancer. (a) The interaction of MEM, COXPRESdb coexpressed genes, and differently expressed genes from TCGA/GTEx data of gastric cancer. Signaling pathways or gene annotations from KEGG (b), BP GO (c), CC GO (d), and MF GO (e). (f) PPI of the genes in the pathway of the cell cycle from KEGG.

**Figure 11:**
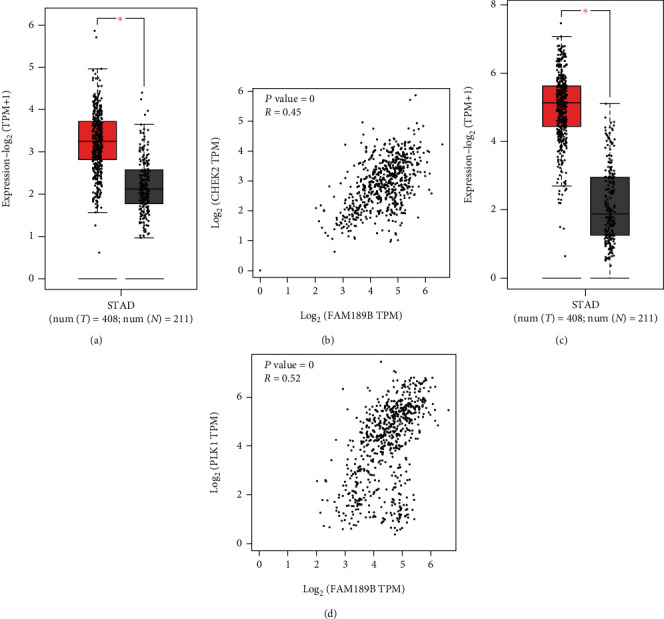
The relationships between FAM189B and two coexpressed genes in gastric cancer. The mRNA levels of CHEK2 (a) and PLK1 (c) based on TCGA/GTEx data. The positive correlations of CHEK2 (b) and PLK1 (d) with FAM189B were assessed by the Pearson analysis.

The interaction of MEM, COXPRESdb coexpressed genes, and DEGs of gastric cancer finally generated 368 genes (Figure 10(a)). KEGG revealed that the pathway of the cell cycle was the top pathway, which included the following coexpressed genes: CDC7, E2F1, CDKN2A, PLK1, TP53, SKP2, PKMYT1, PRKDC, ESPL1, CHEK2, TGFB1, and CDC25B. The first two hub genes of these 12 genes were CHEK2 and PLK1, as shown by the PPI network (Figure 10(f)). Both of the mRNA levels of CHEK2 and PLK1 showed significant upregulation based on TCGA/GTEx data as well as markedly positive relationships with the FAM189B level (Figure 11).

## 4. Discussion

To the best of our knowledge, the mRNA or protein level of FAM189B has never been documented in GC tissues previously. This study revealed that both the mRNA and protein levels of FAM189B were upregulated in the GC samples compared with the noncancerous controls. Furthermore, the increase in FAM189B levels was also positively related to deterioration and an unfavorable outcome in GC, which may be partly due to the correlation with the pathway of the cell cycle.

The physiological and pathological function of FAM189B, which has an official full name of *family with sequence similarity 189 member B* (gene ID: 10712), has not been broadly investigated in different diseases. FAM189B is located at 1q22 with 12 exons, and the ubiquitous expression of FAM189B has been detected in normal fat, testis, and 25 other tissues by the NCBI (https://www.ncbi.nlm.nih.gov/gene/10712). FAM189B has been only rarely studied in various diseases and even less frequently in malignant tumors. So far, only two papers have reported the expression level of FAM189B and its preliminary mechanism in HCC [25, 26]. In GC, there has been no report thus far.

Zhang et al. was the first group to study the clinical role of FAM189B in HCC. They carried out reverse transcription polymerase chain reaction (RT-PCR) and real-time PCR to detect the mRNA expression of FAM189B in HCC samples and cell lines; they also performed immunohistochemical staining to examine the protein expression level of FAM189B. Among 48 HCC cases and 4 invasive HCC cell lines, the expression of both FAM189B mRNA and proteins was upregulated [25]. Later, Zhang et al. enlarged the sample size to 80 pairs of HCC and controls and confirmed the upregulation of FAM189b [26]. In this study, we found for the first time that the expression of FAM189B in GC was similar to that in HCC, as it was also highly expressed in tumor tissue. To understand the clinical potential of FAM189B in GC, we also tested the protein and mRNA levels of FAM189B by various methods in clinical samples. The results showed that FAM189B for both the protein level (immunohistochemistry with tissue microarray) and the mRNA level (technologies of microarray, RNA sequencing, and meta-analysis) revealed consistently significant higher expressions compared with noncancerous gastric tissue. More importantly, the expression data integrated from all sources verified that the SMD of FAM189B expression was 0.46 and the summarized AUC reached 0.79, which highlighted the variation in FAM189B between cancerous and noncancerous tissues. Although a comprehensive analysis with multiple datasets was not possible, the results of several independent studies also indicated that a higher expression of FAM189B was more likely to lead to a malignant progress and a poor prognosis of GC. These results therefore suggest that the higher expression of FAM189B may be a factor that promotes both the occurrence and the clinical deterioration of GC.

Next, we also wanted to identify why the higher expression of FAM189B had an oncogenic capacity in GC. How did it affect the biological function of GC cells? We are the first group to study the clinical significance and molecular mechanism of FAM189B in GC, so there is no record of the effect of FAM189B on cellular growth, apoptosis, invasion, metastasis, or other phenotypes of GC cells. However, the research results of FAM189B in HCC offer some hints. The ectopic transfection of FAM189B improved the *in vitro* cell viability and colony formation of HCC cells in soft agar. Moreover, the *in vivo* tumorigenicity with a tumor xenograft model of HCC-derived cells was also enhanced by the overexpression of FAM189B. In contrast, FAM189B knockdown evidently repressed the cell growth and *in vivo* carcinogenicity in several HCC cell lines [26]. The ectopic transfection of FAM189B also boosted the cell invasion of HCC-derived cell lines LM6 and MHCC-L [25]. As the expression trend of FAM189B in GC was similar to that of HCC, it is very likely that FAM189B will also play an equivalent role in the growth and infiltration of GC cells. Although our *in vitro* experiments have not been completed, we could also see the related pathways from FAM189B in GC from the KEGG and GO analyses, as the pathway of the cell cycle was the top one in the list which was enriched with the FAM189B coexpressed genes in GC.

In HCC, a mechanistic exploration revealed that FAM189B could physically be related to WW domain oxidoreductase (WWOX) and influence WWOX tyrosine phosphorylation. Interestingly, FAM189B ectopic overexpression in HCC Huh7 cells obstructed the WWOX-cyclin D1 pathway, causing cell cycle arrest in HCC YY-8103 cells [25, 26]. In this study, we also showed the clinical significance of two hub genes (CHEK2 and PLK1) in the cell cycle pathway and their correlations with FAM189B. Both of these two coexpressed genes of FAM189B have been well reported to play essential roles in the cell cycle regulation and development of GC [30–34]. Although there was only preliminary evidence of a correlation, this finding also suggests that FAM189B may affect the clinical progression of GC by disturbing the cell cycle of GC cells. Of course, the specific molecular network and exact mechanism will need further study in the future.

This was a preliminary small-scale study on the clinical significance of FAM189B in GC. There were also a few limitations in our study. First, whether FAM189B will aid in diagnosing GC remains to be confirmed by a noninvasive examination method. Second, the mRNA have not been extracted from those samples included in the in-house immunohistochemical test; therefore, the protein and mRNA expression of FAM189B cannot be evaluated in the same sample. Third, our *in vitro* experiment is still in progress. Finally, the specific mechanism of FAM189B also needs further investigation.

## 5. Conclusions

In sum, we have confirmed the overexpressing trend of FAM189B in GC using the expression data of 1274 cases of patients through various detection methods, and FAM189B may also contribute to the progress and poor prognosis of GC. More experiments are underway to answer the remaining questions about its molecular mechanism.

## Figures and Tables

**Table 1:** The protein expression level of FAM189B in gastric cancer tissues.

Clinicopathological parameters	Group	FAM189B expression		*T*	*P* value
		Cases	Mean ± SD		
Tissue	GC tissue	179	10.5978 ± 1.90068		
	Noncancer	147	3.1361 ± 2.42882	30.383	*P* < 0.001

Age	≤60	98	10.4694 ± 1.87882		
	>60	76	10.7105 ± 1.95834	-0.824	0.411

Sex	Male	128	10.5313 ± 1.90704		
	Female	46	10.6957 ± 1.94216	-0.499	0.618

T	T1	3	10.6667 ± 2.3094		
	T2	51	9.451 ± 1.82553		
	T3	120	11.05 ± 1.74823	*F* = 14.466	*P* < 0.001

N	N0	65	9.8308 ± 2.14745		
	N1	87	11.092 ± 1.4518		
	N2	22	10.7273 ± 2.14214	*F* = 8.921	*P* < 0.001

Stage	IA-IB	38	9.2895 ± 1.92996		
	IIA-IIB	117	10.9744 ± 1.66848		
	IIIA	19	10.6842 ± 2.23738	*F* = 12.679	*P* < 0.001

Histological grade	I	28	9.6071 ± 2.31484		
	II	37	10.7568 ± 1.63987		
	III	82	10.939 ± 1.75223	*F* = 3.872	0.011

Among the 179 cases of gastric cancer, some clinical parameters were not provided in five cases, such as age, gender, and TNM stage. In addition, the histological grade information was not provided in 32 cases.

## Data Availability

All data have been checked, and the supplementary explanation of the data is in Table 1.
